# Léiomyosarcome pleural primitif: à propos d'un cas

**DOI:** 10.11604/pamj.2013.15.4.1137

**Published:** 2013-05-03

**Authors:** Imane Ouafki, Tanae Sghiri, Hind El Yacoubi, Saber Boutayeb, Hind Mrabti, Hassan Errihani

**Affiliations:** 1 Service d'Oncologie Médicale, Institut National d'Oncologie (INO), CHU Ibn Sina, Rabat, Maroc

**Keywords:** Léiomyosarcome, plèvre, chimiothérapie, leiomyosarcoma, pleura, chemotherapy

## Abstract

Nous rapportons le cas d'un léiomyosarcome pleural primitif, localement avancé, chez un homme de 64 ans, traité par chimiothérapie. La circonstance de découverte est une masse intra-thoracique, augmentant progressivement de volume, dans un contexte de fièvre et d'altération de l’état général. La tomodensitométrie abdominale a objectivé la tumeur. L'exploration chirurgicale a révélé une tumeur pleurale, très localement avancée, envahissant le médiastin. Une simple biopsie a été réalisée. L'examen anatomopathologique avec complément immunohistochimique était en faveur d'un léiomyosarcome de haut grade. Notre patient a reçu une chimiothérapie à base de Doxorubicine à la dose de 60 mg / m^2^, administrée tous les 21 jours. L’évaluation après 6 cycles de chimiothérapie a retrouvé un bénéfice clinique et une réponse partielle radiologique estimée à 30%. Actuellement, il est en bon contrôle.

## Introduction

Les sarcomes primitifs endothoraciques sont exceptionnels. Ils se développent dans le poumon, le médiastin, la plèvre, et la paroi thoracique. L'angiosarcome, le léiomyosarcome, le rhabdomyosarcome, et le mésothéliome (variante sarcomatoïde) sont les sarcomes primitifs intra thoraciques, les plus fréquents [1]. Le léiomyosarcome primitif thoracique est une tumeur rare et est signalée chez environ 1-4% des patients atteints de sarcomes des tissus mous primitifs de la paroi thoracique [2]. Nous présentons ici un cas de léiomyosarcome pleural primitif localement avancé, traité par chimiothérapie.

## Patient et observation

**Figure 1:**
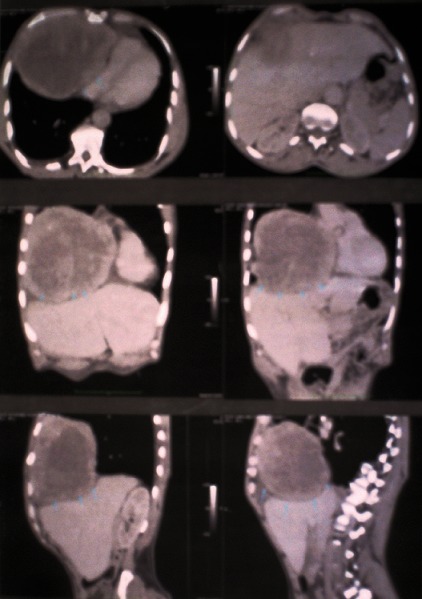
Aspect à la TDM abdominale objectivant un énorme processus lésionnel à paroi épaissie et à centre pseudo-nécrotique, de siège endothoracique, occupant la base thoracique droite, mesurant 14 x 9 cm de grand axe, refoulant la cavité cardiaque avec un discret épanchement péricardique. Cette tumeur a un développement en bas, exerçant un effet de masse sur le segment VIII et IV du foie

Nous rapportons un cas de léiomyosarcome pleural primitif, survenu chez un homme de 64 ans. Le début de sa symptomatologie remonte au mois de Juillet 2010, par l'apparition d'une masse intra thoracique, augmentant progressivement de volume, évoluant dans un contexte de fièvre non chiffrée et d'altération de l′état général. Le patient a été adressé à l'Institut National d'Oncologie (INO), à Rabat, pour prise en charge. L'exploration radiologique a montré sur la tomodensitométrie abdominale un énorme processus lésionnel à paroi épaissie et à centre pseudo-nécrotique, de siège endothoracique, occupant la base thoracique droite, mesurant 14 x 9 cm de grand axe, refoulant la cavité cardiaque avec un discret épanchement péricardique. Cette tumeur a un développement en bas, exerçant un effet de masse sur le segment VIII et IV du foie primitifs (Figure 1). Un complément par angioscanner thoracique a confirmé l'aspect sus décrit. Une thoracotomie à visée diagnostique et thérapeutique a été réalisée. L'exploration chirurgicale a révélé une tumeur pleurale, très localement avancé envahissant le médiastin. Une simple biopsie a été effectuée. L’étude histologique a montré l'existence d'une formation tumorale maligne de nature sarcomateuse, caractérisée par une prolifération de cellules fusiformes disposées en faisceaux irrégulièrement enchevêtrés avec quelques foyers de nécrose ischémique, les cellules montrent une anisonucléose franche avec des noyaux hypertrophiques irréguliers, hyperchromatiques et inconstamment nucléolés, l'activité mitotique est importante (plus de 5 mitoses/10 champs au fort grandissement), un immunomarquage par l'anticorps anti-PS100 est resté négatif, l'anticorps anti-actine muscle lisse montre une positivité focale franche. Le diagnostic de léiomyosarcome de haut grade a été retenu. Notre patient a reçu une chimiothérapie à base de Doxorubicine à la dose de 60 mg / m^2^, administrée tous les 21 jours. L′évaluation après 6 cycles de chimiothérapie a retrouvé un bénéfice clinique et une réponse partielle radiologique estimée à 30%. Actuellement, il est en bon contrôle.

## Discussion

Le léiomyosarcome pleural est une entité rare qui représente 1-4% des sarcomes des tissus mous intra- thoraciques primitifs [2]. Il survient habituellement dans la sixième décennie ou plus tard, avec une prédominance masculine [1]. Cliniquement, il se manifeste par une masse douloureuse rétrosternale, comme il peut être asymptomatique dans les stades précoces [3]. Radiologiquement il apparaît comme un processus nécrotique exerçant un effet de masse sur les structures adjacentes [4]. Le diagnostic initial est souvent difficile dans la mesure où la clinique et l′imagerie ne sont pas spécifiques. Le diagnostic est confirmé par la biopsie et l’étude anatomopatholoqique avec complément immunohistochimique [5]. Pour ce qui est du traitement chirurgical, il est recommandé que la résection doit inclure tous les tissus mous ainsi que les os envahis avec des marges de sécurité suffisantes [6, 7]. En effet ces résections larges de la paroi thoracique contribuent à diminuer les récidives locales, mais sans prolonger la survie [6–8]. Aussi Gordon et al. ont mentionné que les facteurs influençant le pronostic sont le grade histologique de la tumeur et la présence de métastases, mais pas les marges chirurgicales [6]. Cependant, Perry et al. ont rapporté que le facteur le plus important influençant la survie globale est le statut marginal [7].La chimiothérapie est indiquée en cas de léiomyosarcome pleural localement avancé ou métastatique. Elle est essentiellement basée sur l'association doxorubicine à la dose de 60 mg/m^2^ au jour 1 et ifosfamide 5 g/m^2^ au jour 1 administrée tous les 21 jours [1]. Les métastases sont transmises par le sang et sont pour la plupart hépatiques et pulmonaires.

## Conclusion

Le léiomyosarcome pleural est une tumeur extrêmement rare. Son diagnostic est principalement histologique. La chirurgie élargie avec des marges saines reste le traitement de choix. La chimiothérapie a sa place en néo-adjuvant à la chirurgie, ou en cas de maladie métastatique.
